# Narrowband emission enabled by modulation of π-electron delocalization

**DOI:** 10.1093/nsr/nwag312

**Published:** 2026-05-27

**Authors:** Chunyan Chi

**Affiliations:** Department of Chemistry, National University of Singapore, Singapore

Driven by the growing demand for BT.2020-standard ultra-high-definition organic light-emitting diode (OLED) displays, the development of high-performance organic narrowband emitters has attracted tremendous research interest [1]. Among the various candidates, multi-resonance thermally activated delayed fluorescence (MR-TADF) materials, particularly BN-type derivatives, have emerged as one of the most promising classes owing to their intrinsically narrow emission profiles and theoretically 100% internal quantum efficiency [2]. These materials have already enabled remarkable progress in blue and green OLED devices [3,4]. However, similar to conventional fluorescent materials, MR-TADF emitters still face significant challenges in achieving high color purity in the red-emission region, where electroluminescence spectra typically exhibit full width at half-maximum (FWHM) values exceeding 40 nm despite their excellent external quantum efficiencies (EQEs) [3,4]. Therefore, the development of red narrowband emitters remains a critical challenge for next-generation OLED displays.

Theoretical and experimental studies have shown that spectral broadening in organic emitters mainly arises from vibrational coupling and structural relaxation, effects that become particularly pronounced at longer wavelengths [1,5]. This raises a fundamental question: How can vibrational coupling and structural relaxation be effectively suppressed to achieve narrowband emission in organic molecules? In recent work published in *National Science Review*, the team led by Profs You and Bin reported a study on the development of polycyclic aromatic hydrocarbon (PAH)-based narrowband red emitters via modulation of π-electron delocalization [6]. Their molecular design strategy can be viewed as a skeletal reconstruction approach that tunes π-electron delocalization and consequently alters the aromatic character of the molecules. Importantly, they demonstrated that structures

with more localized aromaticity can effectively suppress vibronic coupling, thereby enhancing spectral purity.

**Figure 1.**
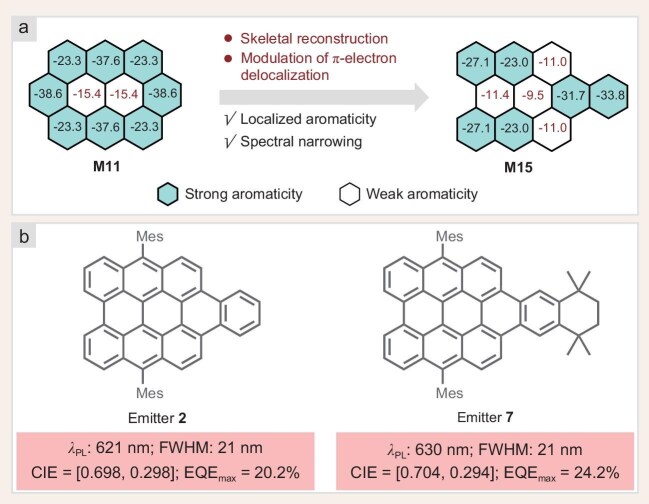
Narrowband emission enabled by modulation of π-electron delocalization. (a) Aromaticity comparison of **M11** and **M15**. The values shown inside the rings represent NICS(1)zz values obtained from Ref. [6]. (b) Chemical structures, solution-phase photophysical properties, and OLED device performances of compounds **2** and **7**.

As a proof-of-concept, the authors systematically reconstructed globally aromatic ovalene (**M11**) into a series of PAHs with diverse fusion modes. Theoretical calculations, including nucleus-independent chemical shift (NICS) analysis and excited-state energy evaluations, revealed that variations in fusion mode can efficiently modulate both molecular aromaticity and emission behavior. On this basis, the **M15** was identified as a promising core structure for further derivatization due to its pronounced localized aromatic character (Fig. 1a). Photophysical studies showed that the **M15**-derived molecular library exhibits excellent emission performance, with FWHM values of 21–24 nm and photoluminescence quantum yields ranging from 62% to 82% at emission wavelengths of 618–630 nm. Representative emitters **2** and **7** were subsequently employed in OLED device fabrication. The optimized devices displayed narrowband electroluminescence with FWHM values below 30 nm, establishing a new benchmark for color purity in red OLEDs. Remarkably, the devices based on emitter **7** exhibited Commission Internationale de l’Éclairage (CIE) coordinates (0.704, 0.294) closely approaching the BT.2020 standard (0.708, 0.292), while also delivering a maximum EQE that ranks among the best reported for conventional fluorescent emitters (Fig. 1b).

In summary, skeletal reconstruction provides a powerful strategy for precise modulation of the electronic properties of π-conjugated frameworks. The intentional disruption of long-range π-electron delocalization effectively suppresses vibrational coupling, thereby enabling narrowband emission. Notably, visualization of the NICS data offers an intuitive interpretation of the mechanism, establishing a clear intrinsic relationship between localized aromaticity and spectral narrowing. This work presents an innovative and practical molecular design strategy for high-performance narrowband emitters and deepens the fundamental understanding of structure−property relationships in fluorescent materials.
